# Genomic analysis and phylogenetic position of the complex IncC plasmid found in the Spanish monophasic clone of *Salmonella enterica* serovar Typhimurium

**DOI:** 10.1038/s41598-021-90299-z

**Published:** 2021-06-01

**Authors:** Xenia Vázquez, Patricia García, Vanesa García, María de Toro, Víctor Ladero, Jürgen J. Heinisch, Javier Fernández, Rosaura Rodicio, M. Rosario Rodicio

**Affiliations:** 1 Departamento de Biología Funcional, Área de Microbiología, Universidad de Oviedo, 33006 Oviedo, Spain; 2 Grupo de Microbiología Traslacional, Instituto de Investigación Sanitaria del Principado de Asturias (ISPA), 33011 Oviedo, Spain; 3 Plataforma de Genómica y Bioinformática, Centro de Investigación Biomédica de La Rioja (CIBIR), 26006 Logroño, Spain; 4 Instituto de Productos Lácteos de Asturias, Consejo Superior de Investigaciones Científicas (IPLA-CSIC), 33300 Villaviciosa, Spain; 5 Grupo de Microbiología Molecular, Instituto de Investigación Sanitaria del Principado de Asturias (ISPA), 33011 Oviedo, Spain; 6 Department of Genetics, Faculty of Biology and Chemistry, University of Osnabrück, Barbarastrasse 11, 49076 Osnabrück, Germany; 7 Servicio de Microbiología, Hospital Universitario Central de Asturias, 33011 Oviedo, Spain; 8 Departamento de Bioquímica y Biología Molecular, Universidad de Oviedo, 33006 Oviedo, Spain; 9 Present Address: Department of Microbiology, University Hospital A Coruña (CHUAC)-Biomedical Research Institute A Coruña (INIBIC), 15006 A Coruña, Spain; 10 Present Address: Laboratorio de Referencia de Escherichia coli (LREC), Departamento de Microbioloxía e Parasitoloxía, Facultade de Veterinaria; Instituto de Investigación Sanitaria de Santiago de Compostela (IDIS), Universidade de Santiago de Compostela (USC), 27002 Lug, Spain

**Keywords:** Evolution, Microbiology

## Abstract

pUO-STmRV1 is an IncC plasmid discovered in the Spanish clone of the emergent monophasic variant of *Salmonella enterica* serovar Typhimurium, which has probably contributed to its epidemiological success. The sequence of the entire plasmid determined herein revealed a largely degenerated backbone with accessory DNA incorporated at four different locations. The acquired DNA constitutes more than two-thirds of the pUO-STmRV1 genome and originates from plasmids of different incompatibility groups, including IncF (such as R100 and pSLT, the virulence plasmid specific of *S*. Typhimurium), IncN and IncI, from the integrative element GI*sul2*, or from yet unknown sources. In addition to pSLT virulence genes, the plasmid carries genes conferring resistance to widely-used antibiotics and heavy metals, together with a wealth of genetic elements involved in DNA mobility. The latter comprise class 1 integrons, transposons, pseudo-transposons, and insertion sequences, strikingly with 14 copies of IS*26*, which could have played a crucial role in the assembly of the complex plasmid. Typing of pUO-STmRV1 revealed backbone features characteristically associated with type 1 and type 2 IncC plasmids and could therefore be regarded as a hybrid plasmid. However, a rooted phylogenetic tree based on core genes indicates that it rather belongs to an ancient lineage which diverged at an early stage from the branch leading to most extant IncC plasmids detected so far. pUO-STmRV1 may have evolved at a time when uncontrolled use of antibiotics and biocides favored the accumulation of multiple resistance genes within an IncC backbone. The resulting plasmid thus allowed the Spanish clone to withstand a wide variety of adverse conditions, while simultaneously promoting its own propagation through vertical transmission.

## Introduction

*Salmonella enterica* serovar 4,[5],12:i:- is a monophasic variant of *S*. Typhimurium, which emerged as a main food-borne pathogen causing outbreaks and sporadic cases of salmonellosis worldwide^1–3^. Several multidrug resistant (MDR) clones of this variant have been described, with the resistance genes being located either on the bacterial chromosome^4–6^ or on plasmids of different incompatibility groups^7,8^. In the so-called “Spanish clone”, the MDR phenotype is conferred by large (ca. 150–200 kb), non-conjugative IncA/C plasmids^7^, here renamed as IncC. This nomenclature follows a proposal based on the compatibility between IncA (formerly IncA/C_1_, represented by plasmid RA1^9^) and IncC (formerly IncA/C_2_) plasmids^10,11^.

Members of the IncC group are large, low-copy number plasmids with broad-host range, and variable capacity for conjugation. The majority of the genes required for conjugation are arranged in two separate regions, namely transfer region 1 and transfer region 2^10,12^. Based on single nucleotide polymorphisms (SNP) in the backbone, IncC plasmids were separated into two major groups, called type 1 and type 2. These two types also differ in Regions R1 and R2, containing alternative versions of an open reading frame located within transfer region 1 (*orf1832* in type 1 and *orf1847* in type 2), and in the large *rhs* (*r*ecombinant *h*ot *s*pot) gene (*rhs1* in type 1 and *rhs2* in type 2). Additionally, two small insertions, i1 (428 bp) and i2 (462 bp), are present in type 2 but not in type 1 plasmids^10,12^. Type 1 plasmids were further separated into type 1a and type 1b, based on SNP accumulations in a 14.5 kb region (Type 1a patch), which starts at the 3’-end of the replication initiation gene *repA* and extends to the region downstream of the *ant*-*tox* antitoxin-toxin genes^13^. Despite incompatibility and entry exclusion, hybrid plasmids with backbone features from both type 1 and type 2, and also with some additional features (like *orf1854* and *rhs3*), have occasionally been found^10^.

IncC plasmids occur in a number of pathogenic bacteria from diverse environments, where they serve as vehicles for classical and emergent resistance genes. Apparently, these genes were acquired multiple times during evolution, usually as part of clearly recognizable mobile genetic elements inserted at particular locations within the IncC backbone^10^. IncC plasmids of the monophasic Spanish clone were previously shown to contain the *bla*_TEM-1_, *cmlA1*, *aac(3)-IV*, [*aadA1*, *aadA2*], [*sul1*, *sul2*, *sul3*], *tet*(A) and *dfrA12* genes which confer resistance to ampicillin, chloramphenicol, gentamicin, streptomycin-spectinomycin, sulfonamides, tetracycline and trimethoprim, respectively^7,14^. Genes potentially encoding resistance to biocides; multiple insertion sequences, transposons and integrons; or derived from the serovar-specific virulence plasmid of *S*. Typhimurium, pSLT, were also detected in the IncC plasmids of the Spanish clone^7,14^.

Given the interest in IncC plasmids as a model to understand the evolutionary mechanisms leading to the acquisition and dissemination of accessory DNA, including not only resistance but also virulence genes, we here decided to determine the complete nucleotide sequence of a plasmid with the IncC backbone (pUO-STmRV1), which was obtained from a clinical isolate selected as a representative of the monophasic Spanish clone of *S*. Typhimurium. Sequence analysis confirmed experimentally obtained results, detected additional resistance genes, revealed novel features related to the structure of the plasmid, and disclosed its phylogenetic position within the IncC group.

## Results and discussion

### pUO-STmRV1 is a complex plasmid shaped by a wealth of genetic elements involved in DNA mobility

**Figure 1:**
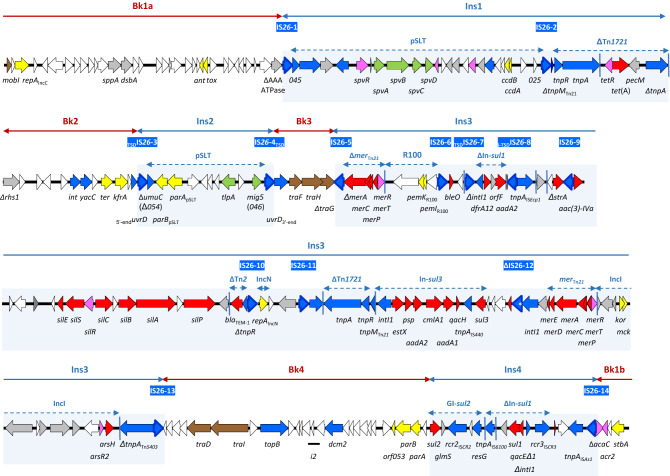
Gene organization of pUO-STmRV1 (drawn to scale from accession number CP018220). Open reading frames are represented by arrows indicating the direction of transcription with different colors based on the predicted functions: yellow, plasmid replication, maintenance and segregation; brown, conjugative transfer; blue, DNA metabolism; light orange, regulation of gene expression; red, resistance; green, virulence; grey, other roles; white, unknown function. The 14 copies of IS*26* detected in pUO-STmRV1 are labelled with white letters on dark blue background. All IS*26* are delineated by inverted repeats (IR; not shown), and all except IS*26*-12 are intact. The frameshift mutation detected in the *tnpA* gene of the latter is indicated with a white asterisk inside the gene. IR in other transposable elements are represented by vertical blue lines. None of the individual IS, including the 14 copies of IS*26*, or Tn are surrounded by target site duplications. The position of the i2 insert (between *topB* and *dcm2*) is indicated by a horizontal black line. The DNA segments (Ins1 to Ins4) inserted into the pUO-STmRV1 backbone are indicated and highlighted within blue boxes. The segments in which the plasmid backbone is distributed (Bk1a and Bk1b, which are contiguous in the circular plasmid, to Bk4), are also indicated.

Plasmid pUO-STmRV1 (Fig. 1) consists of 197,365 bp with a mean GC content of 52.3%. Approximately 170 open reading frames (*orf*) could be identified, comprising all *orfs* of more than 100 nt and some selected smaller *orfs*. Those with predicted functions (62%) were mainly associated with plasmid propagation (replication, maintenance and conjugative transfer), with virulence and resistance, and with multiple genetic elements, including intact or defective insertion sequences (IS*26*, IS*Ecp1*, IS*440*, IS*CR2*, IS*6100*, IS*CR3*, and IS*As1*), transposon remnants (Tn*1721*, Tn*2*, Tn*21* and Tn*5403*), and class 1 integrons of the *sul1* and *sul3* types (Fig. 1). Notably, pUO-STmRV1 harbors 14 copies of IS*26* (IS*26*-1 to IS*26*-14; all intact except IS*26*-12 that has a frameshift mutation in the *tnpA* gene). This insertion sequence has probably played a major role in the reductive evolution observed in the pUO-STmRV1 backbone, as well as in the acquisition of the accessory DNA. IS*26* can provoke these events by (1) generating pseudo-compound transposons (consisting of a central segment flanked by two copies of IS*26* in direct orientation) and translocatable units (formed by one copy of IS*26* and the adjacent DNA), and by (2) using two different mechanisms of movement: (a) the copy-in mechanism, which is replicative since both the IS as well as 8 bp originally present at a randomly selected target site are duplicated, and (b) the targeted conservative mechanism involving two copies of IS*26*, in which IS*26* is neither replicated, nor does the target site duplication (TSD) occur^15–17^. Together with IS*26*, the many other genetic elements found in pUO-STmRV1, and homologous recombination between repeated DNA (like parts of class 1 integrons, of the *mer* operon of Tn*21*, and of Tn*1721*, in addition to IS*26*), could have contributed to the complexity, and also to the high plasticity and intrinsic instability of the IncC plasmid of the monophasic Spanish clone, indicated by the large number of variants discovered so far^7,14^.

### Extensive deletions and loss of synteny altered the IncC backbone in pUO-STmRV1

**Figure 2:**
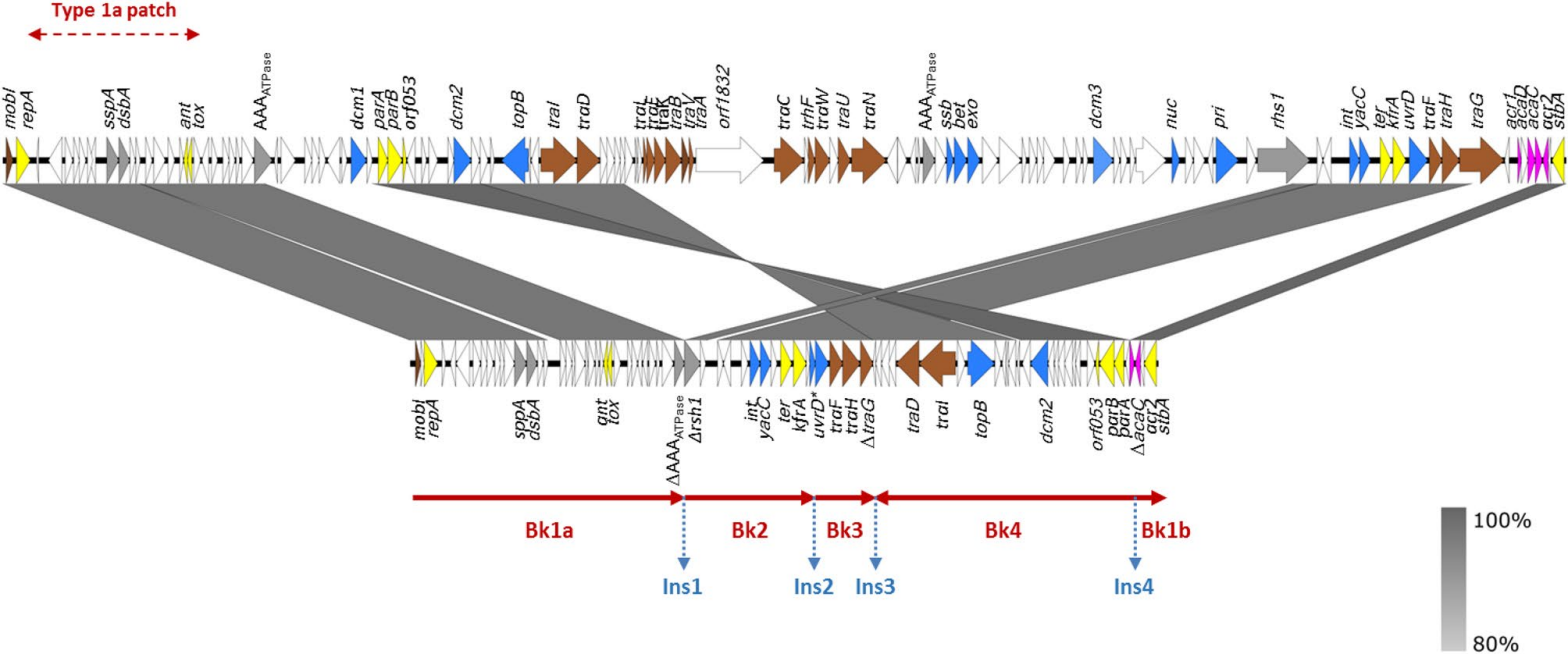
Comparison of the pUO-STmRV1 backbone (below) with that of plasmid pR148 used as reference of the IncC group (above). The alignment was created with EasyFig BLASTn, based on accession no. JX141473 and CP018220. Open reading frames are represented by arrows pointing in the direction of transcription and having different colors based on the predicted functions: yellow, plasmid replication, maintenance and segregation; brown, conjugative transfer; blue, DNA metabolism; light orange, regulation of gene expression; grey, other roles; white, unknown function. Gray shading between the backbones connects regions of nucleotide sequence identity ranging from 80 to 100%, according to the scale shown in the lower right part of the figure. The extent of the four segments of the pUO-STmRV1 backbone (Bk1a and Bk1b, contiguous in the circular plasmid, Bk2, Bk3 and Bk4; the later translocated and inverted with respect to the corresponding segment in pR148), and the position of the insertions located between them (Ins1 to Ins4) are indicated. The *uvrD* gene, interrupted by Ins2 and distributed between Bk2 and Bk3, is marked with an asterisk and represented by two arrows, corresponding to the 5′- and 3′-ends. The type 1a patch used to differentiate type 1a and type 2b IncC plasmids is shown above the pR148 backbone.

Three extensive deletions were detected in the pUO-STmRV1 backbone (Bk), which is considerably smaller than the typical IncC backbone (60,875 bp compared to 127.8 kb and 129.2 kb reported for type 1 and type 2 plasmids, respectively^10^). The remaining backbone is scattered into four segments, here designated as Bk1 to Bk4 (Figs. 1 and 2). The expected order and orientation is conserved for all these segments except Bk4, which corresponds to the *parAB* region^10^ (see below). The 26,987 bp sequence of Bk1 (designated as Bk1a and Bk1b in the linear maps shown in Figs. 1 and 2, but contiguous in the circular plasmid), is flanked by two oppositely oriented copies of IS*26* (IS*26*-14 and IS*26*-1). In addition to *repA*, Bk1a contains the *ant* and *tox* genes (also known as *ata* and *tad*) that encode the antitoxin and toxin of a plasmid addition system, respectively^12,18^. An extensive deletion in Bk1a includes the location of the 428 bp i1 insertion found in type 2 but not in type 1 IncC plasmids. Upstream of *repA*, in Bk1b, the plasmid also lacks sequences of the master regulator required for transcriptional activation of transfer genes^19^, retaining only *acr2* and Δ*acaC*.

With regard to the 10,671 bp Bk2 (Figs. 1 and 2), it is of note that only 1,317 bp of the 3’-end of the *rhs* gene are retained in pUO-STmRV1, and that they share 98% identity to the corresponding end of the *rhs1* gene of the type 1 reference plasmid pR148^10^ (26 mismatches in the common sequence of 1,317 bp). In contrast, only the first 725 bp of the *rhs* remnant of pUO-STmRV1 coincided in the *rhs* genes of R55 and pYR1, showing 93.5% and 94.9% identities, respectively (47 and 37 differences). Deletions in *rhs1* have previously been detected, though they mostly affected the 3’-end of the gene. The insertion site of ARI-A, the resistance island located upstream of *rhs1* in most type 1 plasmids, is missing in pUO-STmRV1, since the deletion affecting the 5’-end of *rhs* extends into the adjacent DNA. Downstream of Δ*rhs*, the *int*, *yacC*, *ter* and *kfrA* genes are followed by the *uvrD* gene, interrupted by an insertion of foreign DNA (see below).

The 4,667 bp Bk3 corresponds to part of transfer region 2, carrying *orfs* homologous to *traF*, *traH* and *traG* (required for assembly of the conjugative type IV secretion system), with *traG* truncated at the 3’-end. Finally, Bk4 comprises 20,890 bp that are translocated and inverted as compared to the typical IncC backbone, causing a loss of synteny (Figs. 1 and 2). Bk4 together with Ins4 (ARI-B; see below) could have been released from Bk1 as part of a larger segment flanked by copies of IS*26* in direct orientation. This will enable the segment to move as a translocatable unit^15,16^, which would have targeted an IS*26* already present at the new location. Intramolecular copy-in transposition by the *trans* pathway^17^, could then have led to the observed inversion, but an additional deletion will be also required to explain the current configuration. Bk4 contains the plasmid partition genes *parA* and *parB*, as well as a conserved *orf* (*orf053*), previously shown to be required for stable plasmid maintenance^18^. This *orf*, together with *repA*, *parA* and *parB*, was selected as part of the pMLST (plasmid multilocus sequence typing) scheme proposed for IncC plasmids^18^. The *traI* and *traD* genes of transfer region 1, which encode a relaxase of the MOB_H_ group^20^ and a coupling protein, respectively, are also located in Bk4. Yet, other transfer genes of this region are absent, as well as any of the two large *orfs* placed downstream of *traA* in type 1 (*orf1832*) and type 2 (*orf1847*) IncC plasmids^10,12^. However, the 462 bp i2 insertion characteristically found in type 2 plasmids is present. Consistent with the extensive deletions affecting the two transfer regions, pUO-STmRV1 failed to be conjugated into *E. coli*^7^. It is finally of note that the RepA, ParA, ParB and Orf053 proteins of pUO-STmRV1, known to be essential for replication and maintenance of IncC plasmids, are all more than 99% identical to the equivalent proteins of pR148, pSN254, R55 and pYR1.

### Nearly two-thirds of pUO-STmRV1 comprises accessory DNA providing virulence genes and genes conferring resistance to antimicrobial agents and heavy metals

Apart from a reduced IncC backbone, pUO-STmRV1 contains four exogenous regions, termed Ins1 to Ins4, which account for 136,525 bp out of the 197,365 bp (69.17%) of the plasmid genome (Figs. 1 and 2). They are located upstream of the truncated *rhs* gene (Ins1), within the *uvrD* gene (Ins2), downstream of Δ*traG* (Ins3), and downstream of *parA* (Ins4). All four are either flanked by or adjacent to IS*26*, with additional copies of the latter appearing interspersed within Ins1 and Ins3. Comparative analysis reveals that large portions of the acquired DNA originated from plasmids of different incompatibility groups, including IncF (such as R100 and pSLT), IncN and IncI, and from the integrative element GI*sul2*^21^. Other sequences are of unknown origin.

Ins1 (30,516 bp) consists of a segment of pSLT-DNA flanked by two copies of IS*26* (IS*26*-1 and IS*26*-2), which is followed by a Δ*tnpM* gene of Tn*21* and a truncated Tn*1721* that supplies the *tetR* and *tet*(A) for tetracycline resistance (Fig. 1). The pSLT DNA (from pSLT045 to pSLT025) comprises the *spv* region, which encodes the main virulence factors associated with serovar-specific virulence plasmids in *S. enterica*^22^, as well as the toxin-antitoxin *ccdAB* genes, which could further enhance the stability of pUO-STmRV1.

Ins2 (10,908 kb), flanked by IS*26*-3 and IS*26*-4, consists of a second segment of pSLT-DNA (from pSLTΔ054 to pSLT046), which comprises the *parAB*_pSLT_ partition genes and the macrophage-induced virulence gene *mig5*, coding for a putative carbonic anhydrase that gets induced inside macrophages. Interestingly, none of the individual copies of IS*26* found in pUO-STmRV1 displays the 8 bp TSD. However, such 8 bp TSD (GTCGAAGG, which belongs to the targeted *uvrD* gene of the IncC backbone) are located at the 5’-end of IS*26*-3 and the 3’-end of IS*26*-4. A two-step mechanism would explain the observed configuration: (1) copy-in transposition of IS*26* into *uvrD* generating the TSD, followed by (2) conservative targeting of this IS element by a translocatable unit^15,16^ consisting of a single copy of IS*26* and the pSLT segment spanning from Δ*umuC* (pSLTΔ054) to *mig5* (pSLT046). This would have occurred after the initial acquisition of a contiguous pSLT segment (from pSLTΔ054 to pSLT025; already carrying an internal copy of IS*26*), which is now separated into Ins1 and Ins2 (Fig. 1).

The large (81,581 bp) and highly complex Ins3 region is also surrounded by two copies of IS*26* (IS*26*-5 and IS*26*-13) and carries seven internal copies of this element (IS*26*-6 to IS*26*-12). Eight bp TSD (TATCTTTA and TAAAGATA; Fig. 1) are only found in the segment flanked by IS*26*-7 and IS*26*-8, although the position and orientation of one of the repeats has been altered. This could have originated from an intramolecular copy-in transposition event by the *trans* pathway^17^, accompanied by inversion of the segment carrying a defective class 1 integron of the *sul1* type. However, inversion of the segment and the TSD through homologous recombination between the oppositely oriented copies of IS*26*, cannot be ruled out. Many other genetic elements and resistance genes are also located within Ins3. They comprise genes involved in resistance to heavy metals (see below), such as arsenic (*arsR2* and *arsH* genes), mercury (with intact: *merRTPCADE* and truncated: *merRTPC*-Δ*merA* copies of the *mer* region of Tn*21*), and silver (*silESRCBAP* genes), as well as additional resistance genes, most of them carried by conventional (*sul1*) and atypical (*sul3*) class 1 integrons, or transposon remnants [∆Tn*2* and ΔTn*1721*]. The *pemI* and *pemK* genes responsible for stable maintenance of plasmid R100, the *repA* gene of the IncN incompatibility group (supplied by a 965 nt segment that is 99.17% identical to the corresponding DNA of R46), and a segment homologous to sequences of IncI plasmids (showing 99.1% identity with pR64 spanning 11.2 kb), including the *kor* and *mck* genes for plasmid stability, as well as the *arsR2* and *arsH* genes, are also located within Ins3. Altogether, four plasmid addition systems are carried by pUO-STmRV1 (*ant*-*tox*, *ccdA*-*ccdB*, *pemK*-*pemI* and *mck*-*kor*), which are likely to ensure maintenance of the plasmid even in the absence of selective pressure. However, this will not prevent further evolution through loss or acquisition of accessory DNA, as a means of adaptation to changing conditions.

Finally, Ins4 (13,520 bp), located downstream of *parA*, is a *sul2*-containing region of the ARI-B type. ARI-B is a resistant island found in both type 1 and type 2 IncC plasmids, which has most likely originated from the integrative element GI*sul2*^21,23^. It covers the region from the *sul2*-end to *resG*, but lacks the other genes of GI*sul2* including the *arsHCB* operon. In contrast, a second class 1 integron of the *sul1* type, can be detected downstream of *resG* which lacks gene cassettes in the variable region but carries *qacEΔ1* and *sul1* in the 3’-conserved segment (Fig. 1).

### pUO-STmRV1 confers resistance to heavy metals

Although many genes carried by pUO-STmRV1 were previously associated with the resistance of LSP 389/97 to multiple antibiotics^7,14^, resistance towards heavy metals has not been experimentally confirmed. According to the presence of the *mer* and *sil* clusters in pUO-STmRV1, LSP 389/97 was resistant to HgCl_2_ and AgNO_3_, with MIC values of 32 µg/ml and 125 µM, respectively, which are 4- to 8- and 4- fold higher than those of the control strains (*S*. Typhimurium LT2 and ATCC 14028; Table 1). In the case of arsenic, toxicity is strongly dependent on the chemical form and oxidation state^24^. It has been demonstrated that the *arsH* gene, which is controlled by the product of *arsR2* (both carried by pUO-STmRV1), encodes an organoarsenical oxidase that detoxifies trivalent methylated and aromatic arsenicals by oxidation to the relatively innocuous pentavalent species^24,25^. Therefore, phenylarsine oxide, an aromatic As(III) compound, was tested in the present study, using the organic As(V) roxarsone, and two inorganic compounds: NaAsO_2_ [As(III)] and Na_2_AsHO_4_ [As(V)], as controls. As expected, the MIC of LSP 389/97 to the inorganic forms (64 µg/ml and 128 µg/ml for NaAsO_2_ and Na_2_AsHO_4_, respectively) coincided with those of the control strains. In contrast, the MIC of the phenylarsine oxide (4 µg/ml), was 16 times higher. In the case of roxarsone, a largely harmless pentavalent organic form, the MIC values were extremely high for the three strains tested. This compound is used in the poultry industry, and to a lesser extent also in the pork industry, as a feed additive to promote growth and prevent coccidial infections^26^. Interestingly, the entire integrative GI*sul2* element carried by plasmid pIP40a and reported as the progenitor of ARI-B, includes an apparently intact *arsRHCB* cluster^23^, which is not present in the deleted ARI-B of pUO-STmRV1. The *Pseudomonas aeruginosa* strain carrying pIP40a was susceptible to NaAsO_2_ and Na_2_HAsO_4_, but resistance to trivalent organic arsenicals was not tested. The products of the *arsR2* and *arsH* genes of pUO-STmRV1 are closely related to those reported in plasmids of the IncI incompatibly group, like R64^27^, but only distantly related to those carried by GI*sul2*.

**Table 1:** Minimum inhibitory concentration (MIC) of heavy metals for *Salmonella enterica* serovar 4,5,12:i:- strain LSP 389/97 carrying plasmid pUO-STmRV1.

Strain	MIC					
	HgCl_2_ (µg/ml)	AgNO_3_ (µM)	NaAsO_2_	Na_2_HAsO_4_ (µg/ml)	Phenylarsine oxide (µg/ml)	Roxarsone (µg/ml)
LSP 389/97	32	125	64	128	4	8,192
LT2	4	31	64	128	0.25	2,048
ATCC 14028	8	31	64	128	0.25	2,048

*S. enterica* serovar Typhimurium strains LT2 and ATCC 14028 were included as negative controls.

### Phylogenetic analysis reveals that pUO-STmRV1 belongs to an ancient lineage which diverged early from the main clade of IncC plasmids

Sequence analysis combined with in silico PCR typing^28^ identified pUO-STmRV1 as a novel hybrid plasmid, sharing features of type 1 IncC plasmids (such as the R2 region containing *rhs1*, although only 1,317 bp of the 3’ end of the gene are conserved in pUO-STmRV1; see above), and type 2 IncC plasmids (i2 insertion). Other specific traits used to define IncC types are missing in pUO-STmRV1, due to the extensive deletions affecting the plasmid backbone (Table 2).

**Table 2:** In silico PCR-typing of pUO-STmRV1.

Plasmid	IncC type	i1	i2	*orf*	*rhs*	ARI-B	Accession number	References
pR148	Type 1a	−	−	1,832	*rhs1*	−	JX141473	^31^
pSN254	Type 1b	−	−	1,832	*rhs1*	+	CP000604	^32^
R55	Type 2	+	+	1,847	*rhs2*	+	JQ010984	^33^
pYR1	Hybrid	+	+	1,847	*rhs3*	+	CP000602	^32^
pUO-STmRV1	Hybrid	− (NP)	+	− (NP)	Δ*rhs1*	+	CP018220	This study

NP, regions surrounding the i1 and *orf* locations are not present; Δ*rhs1,* only 1,317 bp of the 3’-end of *rhs1* (4,254 bp) are present. Based on^28^.

**Figure 3:**
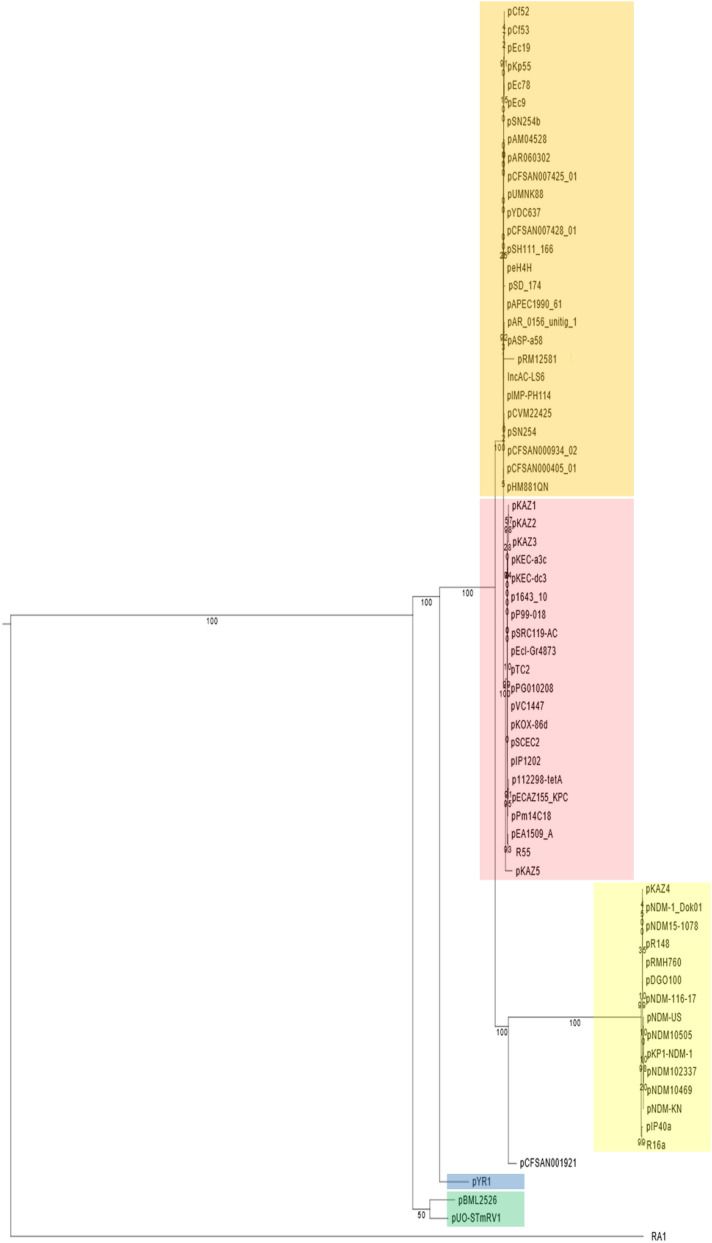
Phylogenetic relationships of pUO-STmRV1. The nucleotide sequences of 67 IncC plasmids and of RA1 (IncA) were extracted from GenBank-NCBI (Table S1). The tree was based on a 17,713 ± 37 bp core genome including 28 orthologous common genes (Table S2) with at least 80% identity and 80% coverage, using 100 bootstrapping replicates. IncC types and subtypes are highlighted in yellow, type 1a; orange, type 1b; pink, type 2; blue, pYR1; green, pUO-STmRV1 and pBML2526. As shown in the pairwise SNP distance matrix used to build the tree (Table S3), the number of SNP separating pUO-STmRV1 from pBML2526, pYR1, pR148 (type 1a), pSN254 (type 1b) and R55 (type 2), which are all IncC, and from the IncA plasmid RA1 are 78, 143, 410, 212, 215 and 1,336, respectively.

To precisely establish the evolutionary position of pUO-STmRV1, a phylogenetic tree was constructed based on SNP detected in a total of 28 core genes conserved in 67 IncC plasmids and RA1 (IncA), separated in the tree as outgroup (Fig. 3; see Tables S1, S2 and S3 for details). pUO-STmRV1 is most closely related to pBML2526, a 204,791 bp plasmid from *Providencia retggeri*. Interestingly, these two plasmids appear to belong to an ancient lineage which diverged at the root of the IncC tree from pYR1 and the main clade including all other IncC plasmids used in the tree. The latter are in turn distributed into two sub-clades. One of them comprises type 1a plasmids and the distantly related pCFSAN001921, previously proposed as a potential new subtype^10^, while the second contains type 1b and type 2 plasmids. The relationship between the latter two groups is not only supported by the numbers of SNP (Table S2), but also by comparisons of the type 1a patch (see Fig. 2) used to discriminate type 1a and type 1b plasmids^10,12^, which showed 99.96% identity between pSN254 (type 1b) and R55 (type 2), but only 96.38% identity between pR148 (type 1a) and pSN254. The pUO-STmRV1 region corresponding to the type 1a patch presented 90.16%, 92.94%, 92.90% and 96.91% identity with the equivalent regions in pR148, pSN254, R55 and pYR1, respectively. It should be noted that with the exception of pYR1, all other IncC plasmids previously reported as hybrids and included in the tree (Table S1), grouped together with either type 1b or type 2 plasmids.

The overall topology of the tree shown in Fig. 3 coincides with that reported by Hancock et al*.*^18^, built in a similar fashion from SNP in core genes using plasmid RA1 as outgroup. Both trees failed to separate type 1 and type 2 plasmids, in contrast to the unrooted tree generated from plasmid backbones, and published by Lei et al*.*^29^. Plasmid backbones are particularly well suited to disclose diversity, since intergenic regions in addition to core genes are used in the alignments, while rooted trees based on SNP differences in core genes are better suited to disclose evolutionary distances.

In conclusion, typing of pUO-STmRV1 reveals backbone features characteristically associated with type 1 and type 2 IncC plasmids, and could thus be regarded as a new hybrid plasmid. Such plasmids are traditionally assumed to originate from homologous recombination between type 1a and type 1b plasmids, despite their incompatibility and entry exclusion mechanisms^11^. However, a phylogenetic analysis based on SNP in core genes suggests that pUO-STmRV1, and also pYR1, belong to ancient lineages which have separated at an early stage from the main branch leading to most extant IncC plasmids detected thus far. pUO-STmRV1 appears to have evolved at a time when uncontrolled use of antibiotics and biocides propitiated the accumulation of multiple resistance genes into an IncC platform. This was facilitated and mediated by a wealth of potentially mobile genetic elements that also provided virulence genes, at the expense of significantly shortening the plasmid backbone. The resulting pUO-STmRV1 allowed the Spanish monophasic clone of *S*. Typhimurium to withstand a variety of adverse conditions, while simultaneously promoting its own maintenance and propagation through accumulation of plasmid addiction systems and by vertical transmission within the clone.

## Methods

### Bacterial isolate, genomic DNA extraction and whole genome sequencing

LSP 389/97 4,5,12:i:- (phage type U302) was the monophasic isolate used in the present study. It was recovered from feces of a patient with gastroenteritis in 1997, and assigned to the Spanish clone^7,14,30^. Genomic DNA of the isolate was extracted with the “GenElute Bacterial Genomic DNA Kit” (Sigma Aldrich) following the manufacturer’s instructions. WGS was performed in parallel with Illumina (short-reads) and PacBio (long-reads) technologies. Illumina sequencing was carried out at Era7 Bioinformatics (Madrid, Spain), using paired-end reads of 90 nt from a fragment library of 500 bp. The reads were assembled with the VelvetOptimizer.pl scrpit implemented in the “on line” version of PLACNETw (https://castillo.dicom.unican.es/upload/). PacBio sequencing was performed at Expression Analysis Inc. (Durham, NC, USA), using the Pacific Biosciences RS II platform, from a library of 6.5 kb DNA fragments on three single-molecule real-time (SMRT) cells (Pacific Biosciences, Menlo Park, CA, USA). The reads were assembled with the Hierarchical Genome Assembly Process (HGAP 3) version 3.0. The assembly comprised two contigs, one corresponding to the entire *Salmonella* chromosome and the other to pUO-STmRV1, which was manually circularized after removing the terminal repeats in the assembled sequence. Errors in the PacBio sequence of pUO-STmRV1 were manually corrected by comparison with Illumina contigs of plasmid origin that were identified with PLACNETw in conjunction with BLASTn comparisons (http://blast.ncbi.nlm.nih.gov/). Moreover, before approaching the sequence of the entire genome of LSP 389/97, plasmid fragments were cloned and subjected to Sanger sequencing (data not shown). In this way, more than 30% of the plasmid sequence (about 60 kb) was already generated, and it was also used to correct errors. The corrected sequence of pUO-STmRV1 is available in GenBank under accession no. CP018220.

### Plasmid annotation, typing and phylogenetic analysis

Annotation of pUO-STmRV1 was automatically accomplished by the NCBI Prokaryotic Genome Annotation Pipeline (http://www.ncbi.nlm.nih.gov/genome/annotation_prok/), and manually refined using sequence comparisons with BLASTn and BLASTp. Typing of the plasmid was performed in silico following the PCR scheme of Harmer and Hall^28^. Plasmids pR148^31^ (accession no. JX141473), pSN254^32^ (CP000604), R55^33^ (JQ010984) and pYR1^32^ (CP000602) were included as representatives of type 1a, type 1b, type 2 and hybrid IncC plasmids, respectively. A phylogenetic tree was constructed based on variable positions (i.e. SNP) in genes encoding core proteins of pUO-STmRV1 and another 67 fully sequenced IncC plasmids, in addition to the IncA plasmid RA1. Plasmid sequences were retrieved from GenBank-NCBI (ftp://ftp.ncbi.nlm.nih.gov/genomes/genbank/bacteria/) and their accession numbers are shown in Table S1. The core genome was defined by the collection of 28 orthologous genes common to all plasmids, which shared at least 80% identity and 80% coverage^34^, and accounted for a core length of 17,713 ± 37 bp (Table S2). The tree was built with RAxML (Randomized Axelerated Maximum Likelihood)^35^, with bootstrap support based on 100 replicates^36^. The pairwise SNP distance matrix used to build the tree is shown in Table S3.

### MICs of heavy metals

To gather additional information on the resistance properties of the monophasic Spanish clone, the minimum inhibitory concentration (MIC) of several heavy metals were determined by microdilution tests using the following compounds and concentrations: HgCl_2_ (0–256 µg/ml); AgNO_3_ (0–125 µM); NaAsO_2_ (0–256 µg/ml); Na_2_HAsO_4_ × 7H_2_O (0–256 µg/ml); phenylarsine oxide [0–16 µg/ml], and roxarsone (4-hydroxy-3-nitrobenzenearsonic acid; 0–8192 µg/ml). All these compounds were purchased from Sigma. *S*. Typhimurium strains LT2 and ATCC 14028 were included as negative controls.

## Supplementary Information

Supplementary Information.
