# Supplementary material for: Genomic analysis and phylogenetic position of the complex IncC plasmid found in the Spanish monophasic clone of Salmonella enterica serovar Typhimurium
Source: Sci Rep. 2021 Jun 1;11:11482. doi: 10.1038/s41598-021-90299-z (PMC8169936; doi:10.1038/s41598-021-90299-z)
Supplement: Supplementary file 1 — Supplementary Information. [file 41598_2021_90299_MOESM1_ESM.pdf]

Table S1. Information on the IncC plasmids (67) and the IncA plasmid (RA1) used for phylogenetic analysis[a]

Plasmid	Size (bp)	Organism	Source	Country	Year of isolation	GenBank accession no.	Reference
				**Type 1 IncC (N=34)**			
pIP40a	167,554	*Pseudomonas aeruginosa*	Human	France	1969	KX709966	1
pDGO100	217,306	*Escherichia coli*	Human	Australia	1981	KU997026	2
pRMH760	170,613	*Klebsiella pneumoniae*	Human	Australia	1997	KF976462	3
pR148	165,906	*Aeromonas hydrophila*	Fish	Thailand	2007	JX141473	4
pNDM 102337	165,974	*E. coli*	Human	Canada	2008	JF714412	5
pNDM-1_Dok01	195,560	*E. coli*	Human	Japan	2009	AP012208	6
pNDM-KN	162,746	*K. pneumoniae*	Human	Kenya	2009	JN157804	7
pNDM10505	166,744	*E. coli*	Human	Canada	2010	JF503991	5
pNDM10469	137,813	*K. pneumoniae*	Human	Canada	2010	JN861072	5
pKP1-NDM-1	137,552	*K. pneumoniae*	Human	Australia	2010	KF992018	8
pNDM-US	140,825	*K. pneumoniae*	Human	USA	2010	CP006661	9
pKAZ4	166,991	Uncultured bacterium	Lake sediment	India	2012	KR827393	10
pNDM-116-17	167,382	*Vibrio cholerae*	Seepage water	India	unk	LN831185	Unpublished
pNDM15-1078	176,048	*E. coli*	Human	Canada	unk	CP012902	11
R16a	170,404	*E. coli*	Human	France	1966	KX156773	12
pAPEC1990 61	161,081	*E. coli*	Turkey	USA	1995	HQ023863	13
pAM04528	158,213	*Salmonella enterica*	Human	USA	1998	FJ621587	14
pSN254	176,473	*S. enterica*	unk	USA	2000	CP000604	15
pSH111_166	165,791	*S. enterica*	Cow	USA	2001	JN983043	16
pAR060302	166,530	*E. coli*	Cow	USA	2002	FJ621588	14
pCFSAN007425_01	166,496	*S. enterica*	Turkey	USA	2002	CP009411	Unpublished
peH4H	148,105	*E. coli*	Cow	USA	2002	FJ621586	14
pCFSAN000934_02	158,521	*S. enterica*	Dog	USA	2003	CP009567	17
pCVM22425	158,195	*S. enterica*	Cow	USA	2003	CP009560	17
pCFSAN000405_01	190,923	*S. enterica*	Turkey	USA	2004	CP009409	Unpublished
IncA/C-LS6	171,925	*S. enterica*	Turkey	USA	2004	JX442976	18
pCFSAN007428_01	164,924	*S. enterica*	Chicken	USA	2006	CP009414	Unpublished
pUMNK88	160,573	*E. coli*	Pig	USA	2007	HQ023862	13
pIMP-PH114	151,885	*K. pneumoniae*	Human	France	2010	KF250428	19
pCFSAN001921	221,009	*S. enterica*	Chicken	USA	2011	CP006050	20
pYDC637	199,469	*E. coli*	Human	USA	2013	KP056256	21
pSN254b	152,216	*A. salmonicida*	Fish	Canada	unk	KJ909290	22
pSD_174	173,673	*S. enterica*	Cow	USA	unk	JF267651	23
pRM12581	64,562	*E. coli*	Lettuce	USA	2010	CP007137	24

**Type 2 IncC (N=17)**							
R55	170,810	*K. pneumoniae*	Human	France	1969	JQ010984	[25]
pIP1202	182,913	*Yersinia pestis*	Human	Madagascar	1995	NC_009141	[15]
pP99-018	150,157	*Photobacterium damselae*	Fish	Japan	1999	NC_008612	[26]
pSRC119-A/C	174,068	*S. enterica*	Pig	Australia	2000	KM670336	[27]
pEA1509_A	162,202	*Enterobacter aerogenes*	Human	France	2001	FO203354	[28]
pPG010208	135,803	*E. coli*	Cow	Chile	2004	NC_019065	[13]
pVC1447	159,635	*V. cholerae*	Human	China	2005	KM083064	[29]
p1643_10	167,779	*S. enterica*	Turkey	Poland	2010	KF056330	[30]
pSCEC2	135,615	*E. coli*	Pig	China	2010	KF152885	[31]
pKAZ1	149,696	Uncultured bacterium	Lake sediment	India	2012	KM506769	[10]
pKAZ2	177,334	Uncultured bacterium	Lake sediment	India	2012	KR827391	[10]
pKAZ3	147,957	Uncultured bacterium	Lake sediment	India	2012	KR827392	[10]
pKEC-a3c	272,297	*Citrobacter freundii*	Environment	USA	2012	CP007558	[32]
pKEC-dc3	268,334	*K. pneumoniae*	Human	USA	2012	CP007732	[32]
pKOX-86d	193,725	*K. oxytoca*	Human	USA	2012	CP008790	Unpublished
pTC2	180,184	*Providencia stuartii*	Human	Greece	2012	JQ824049	[33]
pEcl-Gr4873	153,958	*E. cloacae*	unk	unk	unk	KR559890	[34]
**Type 1/2 hybrid IncC (N=16)**							
pYR1	158,038	*Y. ruckeri*	Fish	USA	unk	CP000602	[15]
pPm14C18	165,992	*Proteus mirabilis*	Chiken	China	2014	KU605240	[35]
pHM881QN	160,687	*K. pneumoniae*	Human	Japan	2008-11	LC055503	[36]
pEc19	188,855	*E. coli*	Gull	Australia	2012	KY887591	[37]
pEc78	233,057	*E. coli*	Gull	Australia	2012	KY887595	[37]
pKp55	215,528	*K. pneumoniae*	Gull	Australia	2012	KY887594	[37]
pCf53	200,459	*C. freundii*	Gull	Australia	2012	KY887593	[37]
pCf52	219,342	*C. freundii*	Gull	Australia	2012	KY887592	[37]
pEc9	228,517	*E. coli*	Gull	Australia	2012	KY887590	[37]
pKAZ5	222,486	Uncultured bacterium	Sediment	India	2012	KR827394	[10]
pASP-a58	198,307	*A. veronii*	Human	USA	2013	CP014775	[38]
pECAZ155_KPC	272,202	*E. coli*	Human	China	2013	CP019001	Unpublished
p11298-tetA	190,310	*C. freundii*	unk	China	<2017	KY986974	[39]
pAR_0156_unitig_1	180,262	*P. mirabilis*	unk	unk	<2017	CP021853	Unpublished
pBML2526	204,791	*Providencia rettgeri*	Human	Japan	2018	LC507075	Unpublished
pUO-STmRV1	197,365	*S. enterica*	Human	Spain	1997	CP018220	This study
**Outgroup (N=1)**							
RA1	143,963	*A. hydrophila*	Fish	Japan	1971	FJ705807	[40]

[a], modified from [32]; N, number of plasmids; unk, unknown.

Supplementary references

1 Harmer, C. J., Hamidian, M. & Hall, R. M. pIP40a, a type 1 IncC plasmid from 1969 carries the integrative element GIsul2 and a novel class II mercury resistance transposon. *Plasmid* **92**, 17-25, doi:10.1016/j.plasmid.2017.05.004 (2017).

2 Harmer, C. J., Partridge, S. R. & Hall, R. M. pDGO100, a type 1 IncC plasmid from 1981 carrying ARI-A and a Tn1696-like transposon in a novel integrating element. *Plasmid* **86**, 38-45, doi:10.1016/j.plasmid.2016.06.002 (2016).

3 Harmer, C. J. & Hall, R. M. pRMH760, a precursor of A/C(2) plasmids carrying bla_{CMY} and bla_{NDM} genes. *Microb Drug Resist* **20**, 416-423, doi:10.1089/mdr.2014.0012 (2014).

4 Del Castillo, C. S. *et al.* Comparative sequence analysis of a multidrug-resistant plasmid from *Aeromonas hydrophila*. *Antimicrob Agents Chemother* **57**, 120-129, doi:10.1128/AAC.01239-12 (2013).

5 Mulvey, M. R., Grant, J. M., Plewes, K., Roscoe, D. & Boyd, D. A. New Delhi metallo-beta-lactamase in *Klebsiella pneumoniae* and *Escherichia coli,* Canada. *Emerg Infect Dis* **17**, 103-106, doi:10.3201/eid1701.101358 (2011).

6 Sekizuka, T. *et al.* Complete sequencing of the $bla_{(NDM-1)}$-positive IncA/C plasmid from *Escherichia coli* ST38 isolate suggests a possible origin from plant pathogens. *PLoS One* **6**, e25334, doi:10.1371/journal.pone.0025334 (2011).

7 Carattoli, A., Villa, L., Poirel, L., Bonnin, R. A. & Nordmann, P. Evolution of IncA/C $blaC_{MY-(2)}$-carrying plasmids by acquisition of the $bla_{NDM-(1)}$ carbapenemase gene. *Antimicrob Agents Chemother* **56**, 783-786, doi:10.1128/AAC.05116-11 (2012).

8 Wailan, A. M. *et al.* Mechanisms involved in acquisition of bla_{NDM} genes by IncA/C2 and IncFIIY plasmids. *Antimicrob Agents Chemother* **60**, 4082-4088, doi:10.1128/AAC.00368-16 (2016).

9 Hudson, C. M., Bent, Z. W., Meagher, R. J. & Williams, K. P. Resistance determinants and mobile genetic elements of an NDM-1-encoding *Klebsiella pneumoniae* strain. *PLoS One* **9**, e99209, doi:10.1371/journal.pone.0099209 (2014).

10 Flach, C. F. *et al.* Isolation of novel IncA/C and IncN fluoroquinolone resistance plasmids from an antibiotic-polluted lake. *J Antimicrob Chemother* **70**, 2709-2717, doi:10.1093/jac/dkv167 (2015).

11 Mataseje, L. F. *et al.* Colistin-nonsusceptible *Pseudomonas aeruginosa* sequence type 654 with bla_{NDM-1} arrives in North America. *Antimicrob Agents Chemother* **60**, 1794-1800, doi:10.1128/AAC.02591-15 (2016).

12 Szabo, M. *et al.* Characterization of two multidrug-resistant IncA/C plasmids from the 1960s by using the MinION sequencer device.

Antimicrob Agents Chemother **60**, 6780-6786, doi:10.1128/AAC.01121-16 (2016).

13 Fernandez-Alarcon, C., Singer, R. S. & Johnson, T. J. Comparative genomics of multidrug resistance-encoding IncA/C plasmids from commensal and pathogenic *Escherichia coli* from multiple animal sources. *PLoS One* **6**, e23415, doi:10.1371/journal.pone.0023415 (2011).

14 Call, D. R. *et al.* bla_{CMY-2}-positive IncA/C plasmids from *Escherichia coli* and *Salmonella enterica* are a distinct component of a larger lineage of plasmids. *Antimicrob Agents Chemother* **54**, 590-596, doi:10.1128/AAC.00055-09 (2010).

15 Welch, T. J. *et al.* Multiple antimicrobial resistance in plague: an emerging public health risk. *PLoS One* **2**, e309, doi:10.1371/journal.pone.0000309 (2007).

16 Han, J. *et al.* DNA sequence analysis of plasmids from multidrug resistant *Salmonella enterica* serotype Heidelberg isolates. *PLoS One* **7**, e51160, doi:10.1371/journal.pone.0051160 (2012).

17 Cao, G. *et al.* Complete sequences of sx IncA/C plasmids of multidrug-resistant *Salmonella enterica* subsp. enterica serotype Newport. *Genome Announc* **3**, doi:10.1128/genomeA.00027-15 (2015).

18 Villa, L. *et al.* Reversion to susceptibility of a carbapenem-resistant clinical isolate of *Klebsiella pneumoniae* producing KPC-3. *J Antimicrob Chemother* **68**, 2482-2486, doi:10.1093/jac/dkt235 (2013).

19 Ho, P. L. *et al.* pIMP-PH114 carrying bla_{IMP-4} in a *Klebsiella pneumoniae* strain is closely related to other multidrug-resistant IncA/C2 plasmids. *Curr Microbiol* **68**, 227-232, doi:10.1007/s00284-013-0471-x (2014).

20 Hoffmann, M. *et al.* Complete genome sequence of a multidrug-resistant *Salmonella enterica* Serovar Typhimurium var. 5- strain Isolated from chicken breast. *Genome Announc* **2**, doi:10.1128/genomeA.00294-14 (2014).

21 Lee, C. S., Li, J. J. & Doi, Y. Complete sequence of conjugative IncA/C plasmid encoding CMY-2 beta-lactamase and RmtE 16S rRNA methyltransferase. *Antimicrob Agents Chemother* **59**, 4360-4361, doi:10.1128/AAC.00852-15 (2015).

22 Vincent, A. T. *et al.* Detection of variants of the pRAS3, pAB5S9, and pSN254 plasmids in *Aeromonas salmonicida* subsp. salmonicida: multidrug resistance, interspecies exchanges, and plasmid reshaping. *Antimicrob Agents Chemother* **58**, 7367-7374, doi:10.1128/AAC.03730-14 (2014).

23 Han, J., Lynne, A. M., David, D. E., Nayak, R. & Foley, S. L. Sequencing of plasmids from a multi-antimicrobial resistant *Salmonella enterica* serovar Dublin strain. *Food Research International* **45**, 931-934 (2012).

24 Cooper, K. K. *et al.* Complete genome sequences of two *Escherichia coli* O145:H28 outbreak strains of food origin. *Genome Announc* **2**, doi:10.1128/genomeA.00482-14 (2014).

25 Doublet, B. *et al.* Complete nucleotide sequence of the multidrug resistance IncA/C plasmid pR55 from *Klebsiella pneumoniae* isolated in 1969. *J Antimicrob Chemother* **67**, 2354-2360, doi:10.1093/jac/dks251 (2012).

26 Kim, M. J. *et al.* Complete DNA sequence and analysis of the transferable multiple-drug resistance plasmids (R Plasmids) from *Photobacterium damselae* subsp. piscicida isolates collected in Japan and the United States. *Antimicrob Agents Chemother* **52**, 606-611, doi:10.1128/AAC.01216-07 (2008).

27 Harmer, C. J., Holt, K. E. & Hall, R. M. A type 2 A/C2 plasmid carrying the *aacC4* apramycin resistance gene and the *erm(42)* erythromycin resistance gene recovered from two *Salmonella enterica* serovars. *J Antimicrob Chemother* **70**, 1021-1025, doi:10.1093/jac/dku489 (2015).

28 Diene, S. M. *et al.* The rhizome of the multidrug-resistant *Enterobacter aerogenes* genome reveals how new "killer bugs" are created because of a sympatric lifestyle. *Mol Biol Evol* **30**, 369-383, doi:10.1093/molbev/mss236 (2013).

29 Wang, R. *et al.* IncA/C plasmids harboured in serious multidrug-resistant *Vibrio cholerae* serogroup O139 strains in China. *Int J Antimicrob Agents* **45**, 249-254, doi:10.1016/j.ijantimicag.2014.10.021 (2015).

30 Wasyl, D., Kern-Zdanowicz, I., Domanska-Blicharz, K., Zajac, M. & Hoszowski, A. High-level fluoroquinolone resistant *Salmonella enterica* serovar Kentucky ST198 epidemic clone with IncA/C conjugative plasmid carrying $bla_{(CTX\text{-}M\text{-}25)}$ gene. *Vet Microbiol* **175**, 85-91, doi:10.1016/j.vetmic.2014.10.014 (2015).

31 Zhang, W. J. *et al.* Characterization of the IncA/C plasmid pSCEC2 from Escherichia coli of swine origin that harbours the multiresistance gene cfr. *J Antimicrob Chemother* **69**, 385-389, doi:10.1093/jac/dkt355 (2014).

32 Conlan, S. *et al.* Single-molecule sequencing to track plasmid diversity of hospital-associated carbapenemase-producing Enterobacteriaceae. *Sci Transl Med* **6**, 254ra126, doi:10.1126/scitranslmed.3009845 (2014).

33 Drieux, L. *et al.* Complete nucleotide sequence of the large conjugative pTC2 multireplicon plasmid encoding the VIM-1 metallo-beta-lactamase. *J Antimicrob Chemother* **68**, 97-100, doi:10.1093/jac/dks367 (2013).

34 Papagiannitsis, C. C. *et al.* Characterisation of IncA/C2 plasmids carrying an *In416*-like integron with the $bla_{VIM\text{-}19}$ gene from *Klebsiella pneumoniae* ST383 of Greek origin. *Int J Antimicrob Agents* **47**, 158-162, doi:10.1016/j.ijantimicag.2015.12.001 (2016).

35 Lei, C. W. *et al.* A novel type 1/2 hybrid IncC plasmid carrying fifteen antimicrobial resistance genes recovered from *Proteus mirabilis* in China. *Plasmid* **93**, 1-5, doi:10.1016/j.plasmid.2017.07.002 (2017).

36 Okade, H. *et al.* Characterization of plasmid-mediated quinolone resistance determinants in *Klebsiella pneumoniae* and *Escherichia coli* from Tokai, Japan. *J Infect Chemother* **20**, 778-783, doi:10.1016/j.jiac.2014.08.018 (2014).

37 Papagiannitsis, C. C., Kutilova, I., Medvecky, M., Hrabak, J. & Dolejska, M. Characterization of the complete nucleotide sequences of IncA/C2 plasmids carrying In809-like integrons from Enterobacteriaceae isolates of wildlife origin. *Antimicrob Agents Chemother* **61**, doi:10.1128/AAC.01093-17 (2017).

38 Hughes, H. Y. *et al.* Detection and whole-genome sequencing of carbapenemase-producing *Aeromonas hydrophila* isolates from routine perirectal surveillance culture. *J Clin Microbiol* **54**, 1167-1170, doi:10.1128/JCM.03229-15 (2016).

39 Ma, L. *et al.* Comparative genomics of type 1 IncC plasmids from China. *Future Microbiol* **12**, 1511-1522, doi:10.2217/fmb-2017-0072 (2017).

40 Fricke, W. F. *et al.* Comparative genomics of the IncA/C multidrug resistance plasmid family. *J Bacteriol* **191**, 4750-4757, doi:10.1128/JB.00189-09 (2009).

Table S2. Core genes conserved in the plasmids used to construct the phylogenetic tree[a]

Gene number[b]<br>GenBank (Prodigal)	Product	Gene position[c]	Length (bp)<br>GenBank (Prodigal)[d]
A5895_25800 (100)	Hypothetical protein	88,931-88,425c	507
A5895_25805 (101)	Thymidylate kinase	89,598-88,924c	675
Not annotated (103)[e]	Hypothetical protein	90,543-90,686	144
A5895_25825 (105)	Putative DNA binding protein Orf053	91,738-91,466c	273
A5895_25830 (106)	Plasmid partitioning protein ParB	92,968-91,787c	1182
A5895_25835 (107)	Plasmid partitioning protein ParA	93,757-92,972c	786
A5895_25840 (108)	Hypothetical protein	94,242-93,931c	312
A5895_25970 (124)	DNA binding protein	108,607-108,188c	420
A5895_25975 (125)	Hypothetical protein	108,902-108,609c	294
A5895_25980 (126)	Plasmid stability protein StbA	109,902-108,919c	984
A5895_25985 (127)	Hypothetical protein	110,401-110,940	540
A5895_25990 (128)	Hypothetical protein	110,945-111,232	288
A5895_25995 (129)	Plasmid replication protein RepA	111,217-112,317	1,101 (1,065; 111,253)
A5895_26010 (131)	Hypothetical protein	113,789-113,355c	435
A5895_26020 (133)	Hypothetical protein	115,386-115,799	414 (609; 115,191)
A5895_26025 (134)	Hypothetical protein	115,804-116,325	522
A5895_26030 (135)	Hypothetical protein	116,328-116,849	522
A5895_26035 (136)	Hypothetical protein	116,976-117,419	444 (474; 116,946)
A5895_26040 (137)	Hypothetical protein	117,409-117,723	315 (294; 117,430)
A5895_26045 (138)	Hypothetical protein	117,728-118,582	855
A5895_26050 (139)	S49 family peptidase SspA	118,564-119,541	978 (960; 118,582)
A5895_26055 (140)	Protein disulfide isomerase DbsA	119,557-120,417	861
A5895_26085 (146)	Hypothetical protein	123,263-123,490	228
A5895_26090 (147)	Hypothetical protein	123,477-124,334	858
A5895_26105 (150)	Hipothetical protein	125,150-125,422	273
A5895_26415 (210)	DNA replication terminus site-binding protein Ter	170,745-171,620	876
A5895_26505 (228)	Conjugal transfer protein TraF	185,638-186,678	1041 (1,029; 185,650)
A5895_26510 (229)	Conjugal transfer protein TraH	186,680-188,113	1434

[a], The core genome was defined as the collection of orthologous genes common to all plasmids, which shared at least 80% identity and 80% coverage. They were predicted with Software Prodigal 2.6.3.

[b], Gene numbers according to GenBank (accession number CP018220), and Prodigal (shown in parenthesis).

[c], Gene position according to GenBank; c, denotes gene in the complementary strand.

[d], When GenBank and Prodigal annotations did not exactly coincided, the size of the gene determined by Prodigal is shown in parenthesis; followed by the alternative start site responsible for the difference.

	p112298-tetA	p1643_10	pAM04528	pAPEC1990_61	pAR060302	pAR_0156_unitig_1	pASP-a58	pBML2526	pCFSAN000405_01	pCFSAN000934_02	pCFSAN001921	pCFSAN007425_01	pCFSAN007428_01	pCVM22425	pCf52	pCf53	pDGO100	pEA1509_A	pECAZ155_KPC	pEc19	pEc78	pEc9	pEcl-Gr4873	pHM881QN	pIMP-PH114	pIP1202	pIP40a	IncAC-LS6	pKAZ1	pKAZ2	pKAZ3	pKAZ4	pKAZ5	pKEC-a3c
p112298-tetA		2	10	9	10	10	10	216	9	10	61	10	9	10	10	10	272	3	0	10	10	10	2	9	9	2	273	9	4	4	5	272	17	3
p1643_10	2		8	7	8	8	8	214	7	8	59	8	7	8	8	8	270	1	2	8	8	8	0	7	7	0	271	8	2	2	3	270	15	1
pAM04528	10	8		1	0	2	2	216	1	0	53	0	1	0	2	2	264	9	10	2	2	2	8	1	1	8	265	2	10	10	11	264	17	9
pAPEC1990_61	9	7	1		1	1	1	215	0	1	52	1	0	1	1	1	263	8	9	1	1	1	7	0	0	7	264	1	9	9	10	263	16	8
pAR060302	10	8	0	1		2	2	216	1	0	53	0	1	0	2	2	264	9	10	2	2	2	8	1	1	8	265	2	10	10	11	264	17	9
pAR_0156_unitig_1	10	8	2	1	2		0	216	1	2	53	2	1	2	2	2	264	9	10	2	2	2	8	1	1	8	265	2	10	10	11	264	17	9
pASP-a58	10	8	2	1	2	0		216	1	2	53	2	1	2	2	2	264	9	10	2	2	2	8	1	1	8	265	2	10	10	11	264	17	9
pBML2526	216	214	216	215	216	216	216		215	216	219	216	215	216	216	216	418	215	216	216	216	216	214	215	215	214	421	216	216	216	217	418	221	215
pCFSAN000405_01	9	7	1	0	1	1	1	215		1	52	1	0	1	1	1	263	8	9	1	1	1	7	0	0	7	264	1	9	9	10	263	16	8
pCFSAN000934_02	10	8	0	1	0	2	2	216	1		53	0	1	0	2	2	264	9	10	2	2	2	8	1	1	8	265	2	10	10	11	264	17	9
pCFSAN001921	61	59	53	52	53	53	53	219	52	53		53	52	53	53	53	243	60	61	53	53	53	59	52	52	59	244	53	61	61	62	243	68	60
pCFSAN007425_01	10	8	0	1	0	2	2	216	1	0	53		1	0	2	2	264	9	10	2	2	2	8	1	1	8	265	2	10	10	11	264	17	9
pCFSAN007428_01	9	7	1	0	1	1	1	215	0	1	52	1		1	1	1	263	8	9	1	1	1	7	0	0	7	264	1	9	9	10	263	16	8
pCVM22425	10	8	0	1	0	2	2	216	1	0	53		1		2	2	264	9	10	2	2	2	8	1	1	8	265	2	10	10	11	264	17	9
pCf52	10	8	2	1	2	2	2	216	1	2	53	2	1	2		0	264	9	10	0	0	0	8	1	1	8	265	2	10	10	11	264	17	9
pCf53	10	8	2	1	2	2	2	216	1	2	53	2	1	2			264	9	10	0	0	0	8	1	1	8	265	2	10	10	11	264	17	9
pDGO100	272	270	264	263	264	264	264	418	263	264	243	264	263	264	264	264		271	272	264	264	264	270	263	263	270	5	264	272	272	273	0	279	271
pEA1509_A	3	1	9	8	9	9	9	215	8	9	60	9	8	9	9	9	271		3	9	9	9	1	8	8	1	272	9	3	3	4	271	16	2
pECAZ155_KPC	0	2	10	9	10	10	10	216	9	10	61	10	9	10	10	10	272	3		10	10	10	2	9	9	2	273	9	4	4	5	272	17	3
pEc19	10	8	2	1	2	2	2	216	1	2	53	2	1	2	0	0	264	9	10		0	0	8	1	1	8	265	2	10	10	11	264	17	9
pEc78	10	8	2	1	2	2	2	216	1	2	53	2	1	2	0	0	264	9	10	0		0	8	1	1	8	265	2	10	10	11	264	17	9
pEc9	10	8	2	1	2	2	2	216	1	2	53	2	1	2	0	0	264	9	10	0	0		8	1	1	8	265	2	10	10	11	264	17	9
pEcl-Gr4873	2	0	8	7	8	8	8	214	7	8	59	8	7	8	8	8	270	1	2	8	8	8		7	7	0	271	8	2	2	3	270	15	1
pHM881QN	9	7	1	0	1	1	1	215	0	1	52	1	0	1	1	1	263	8	9	1	1	1	7		0	7	264	1	9	9	10	263	16	8
pIMP-PH114	9	7	1	0	1	1	1	215	0	1	52	1	0	1	1	1	263	8	9	1	1	1	7	0		7	264	1	9	9	10	263	16	8
pIP1202	2	0	8	7	8	8	8	214	7	8	59	8	7	8	8	8	270	1	2	8	8	8	0	7	7		271	8	2	2	3	270	15	1
pIP40a	273	271	265	264	265	265	265	421	264	265	244	265	264	265	265	265	5	272	273	265	265	265	271	264	264	271		265	273	273	274	5	280	272
IncAC-LS6	9	8	2	1	2	2	2	216	1	2	53	2	1	2	2	2	264	9	9	2	2	2	8	1	1	8	265		10	10	11	264	17	9
pKAZ1	4	2	10	9	10	10	10	216	9	10	61	10	9	10	10	10	272	3	4	10	10	10	2	9	9	2	273	10		0	1	272	17	3
pKAZ2	4	2	10	9	10	10	10	216	9	10	61	10	9	10	10	10	272	3	4	10	10	10	2	9	9	2	273	10	0		1	272	17	3
pKAZ3	5	3	11	10	11	11	11	217	10	11	62	11	10	11	11	11	273	4	5	11	11	11	3	10	10	3	274	11	1	1		273	18	4
pKAZ4	272	270	264	263	264	264	264	418	263	264	243	264	263	264	264	264	0	271	272	264	264	264	270	263	263	270	5	264	272	272	273		279	271
pKAZ5	17	15	17	16	17	17	17	221	16	17	68	17	16	17	17	17	279	16	17	17	17	17	15	16	16	15	280	17	17	17	18	279		16
pKEC-a3c	3	1	9	8	9	9	9	215	8	9	60	9	8	9	9	9	271	2	3	9	9	9	1	8	8	1	272	9	3	3	4	271	16	
pKEC-dc3	3	1	9	8	9	9	9	215	8	9	60	9	8	9	9	9	271	2	3	9	9	9	1	8	8	1	272	9	3	3	4	271	16	0
pKOX-86d	2	0	8	7	8	8	8	214	7	8	59	8	7	8	8	8	270	1	2	8	8	8	0	7	7	0	271	8	2	2	3	270	15	1
pKP1-NDM-1	274	272	266	265	266	266	266	420	265	266	245	266	265	266	266	266	2	273	274	266	266	266	272	265	265	272	7	266	274	274	275	2	281	273
pKp55	10	8	2	1	2	2	2	216	1	2	53	2	1	2	0	0	264	9	10	0	0	0	8	1	1	8	265	2	10	10	11	264	17	9
pNDM-116-17	272	270	264	263	264	264	264	418	263	264	243	264	263	264	264	264	0	271	272	264	264	264	270	263	263	270	5	264	272	272	273	0	279	271
pNDM-1_Dok01	272	270	264	263	264	264	264	418	263	264	243	264	263	264	264	264	0	271	272	264	264	264	270	263	263	270	5	264	272	272	273	0	279	271
pNDM-KN	274	272	266	265	266	266	266	420	265	266	245	266	265	266	266	266	2	273	274	266	266	266	272	265	265	272	7	266	274	274	275	2	281	273
pNDM-US	274	272	266	265	266	266	266	420	265	266	245	266	265	266	266	266	2	273	274	266	266	266	272	265	265	272	7	266	274	274	275	2	281	273
pNDM102337	274	272	266	265	266	266	266	420	265	266	245	266	265	266	266	266	2	273	274	266	266	266	272	265	265	272	7	266	274	274	275	2	281	273
pNDM10469	274	272	266	265	266	266	266	420	265	266	245	266	265	266	266	266	2	273	274	266	266	266	272	265	265	272	7	266	274	274	275	2	281	273
pNDM10505	274	272	266	265	266	266	266	420	265	266	245	266	265	266	266	266	2	273	274	266	266	266	272	265	265	272	7	266	274	274	275	2	281	273
pNDM15-1078	272	270	264	263	264	264	264	418	263	264	243	264	263	264	264	264	0	271	272	264	264	264	270	263	263	270	5	264	272	272	273	0	279	271
pP99-018	2	0	8	7	8	8	8	214	7	8	59	8	7	8	8	8	270	1	2	8	8	8	0	7	7	0	271	8	2	2	3	270	15	1
pPG010208	2	0	8	7	8	8	8	214	7	8	59	8	7	8	8	8	270	1	2	8	8	8	0	7	7	0	271	8	2	2	3	270	15	1
pPm14C18	1	1	9	8	9	9	9	215	8	9	60	9	8	9	9	9	271	2	1	9	9	9	1	8	8	1	272	9	3	3	4	271	16	2
pR148	272	270	264	263	264	264	264	418	263	264	243	264	263	264	264	264	0	271	272	264	264	264	270	263	263	270	5	264	272	272	273	0	279	271
R16a	271	269	263	262	263	263	263	419	262	263	242	263	262	263	263	263	3	270	271	263	263	263	269	262	262	269	2	263	271	271	272	3	278	270
R55	3	1	9	8	9	9	9	215	8	9	60	9	8	9	9	9	271	0	3	9	9	9	1	8	8	1	272	9	3	3	4	271	16	2
RA1	1368	1366	1366	1365	1366	1366	1366	1338	1365	1366	1365	1366	1365	1366	1366	1366	1426	1367	1368	1366	1366	1366	1366	1365	1365	1366	1425	1366	1368	1368	1369	1426	1374	1365
pRM12581	9	7	1	0	1	1	1	215	0	1	52	1	0	1	1	1	263	8	9	1	1	1	7	0	0	7	264	1	9	9	10	263	16	8
pRMH760	272	270	264	263	264	264	264	418	263	264	243	264	263	264	264	264	0	271	272	264	264	264	270	263	263	270	5	264	272	272	273	0	279	271
pSCEC2	2	0	8	7	8	8	8	214	7	8	59	8	7	8	8	8	270	1	2	8	8	8	0	7	7	0	271	8	2	2	3	270	15	1
pSD_174	12	10	4	3	4	4	4	218	3	4	55	4	3	4	4	4	266	11	12	4	4	4	10	3	3	10	267	4	12	12	13	266	19	11
pSH111_166	9	7	1	0	1	1	1	215	0	1	52	1	0	1	1	1	263	8	9	1	1	1	7	0	0	7	264	1	9	9	10	263	16	8
pSN254	10	8	0	1	0	2	2	216	1	0	53	0	1	0	2	2	264	9	10	2	2	2	8	1	1	8	265	2	10	10	11	264	17	9
pSN254b	9	7	1	0	1	1	1	215	0	1	52	1	0	1	1	1	263	8	9	1	1	1	7	0	0	7	264	1	9	9	10	263	16	8
pSRC119-AC	3	1	9	8	9	9	9	215	8	9	60	9	8	9	9	9	271	2	3	9	9	9	1	8	8	1	272	9	3	3	4	271	16	2
pTC2	2	0	8	7	8	8	8	214	7	8	59	8	7	8	8	8	270	1	2	8	8	8	0	7	7	0	271	8	2	2	3	270	15	1
pUMNK88	10	8	2	1	2	2	2	216	1	2	53	2	1	2	2	2	264	9	10	2	2	2	8	1	1	8	265	2	10	10	11	264	17	9
pUO-STmRV1	216	214	212	211	212	212	212	76	211	212	211	212	211	212	212	212	410	215	216	212	212	212	214	211	211	214	413	212	216	216	217	410	223	215
pVC1447	2	0	8	7	8	8	8	214	7	8	59	8	7	8	8	8	270	1	2	8	8	8	0	7	7	0	271	8	2	2	3	270	15	1
pYDC637	9	7	1	0	1	1	1	215	0	1	52	1	0	1	1	1	263	8	9	1	1	1	7	0	0	7	264	1	9	9	10	263	16	8
pYR1	153	151	147	146	147	147	147	156	146	147	176	147	146	147	147	147	374	152	153	147	147	147	151	146	146	151	375	147	153	153	154	374	160	152
peH4H	9	7	1	0	1	1	1	215	0	1	52	1	0	1	1	1	263	8	9	1	1	1	7	0	0	7	264	1	9	9	10	263	16	8

	pKEC-dc3	pKOX-86d	pKP1-NDM-1	pKp55	pNDM-116-17	pNDM-1_Dok01	pNDM-KN	pNDM-US	pNDM102337	pNDM10469	pNDM10505	pNDM15-1078	pP99-018	pPG010208	pPm14C18	pR148	R16a	R55	RA1	pRM12581	pRMH760	pSCEC2	pSD_174	pSH111_166	pSN254	pSN254b	pSRC119-AC	pTC2	pUMNK88	pUO-STmRV1	pVC1447	pYDC637	pYR1	peH4H
p112298-tetA	3	2	274	10	272	272	274	274	274	274	274	272	2	2	1	272	271	3	1368	9	272	2	12	9	10	9	3	2	10	216	2	9	153	9
p1643_10	1		272	8	270	270	272	272	272	272	272	270	0	0	1	270	269	1	1366	7	270	0	10	7	8	7	1	0	8	214	0	7	151	7
pAM04528	9	8	266	2	264	264	266	266	266	266	266	264	8	8	9	264	263	9	1366	1	264	8	4	1	0	1	9	8	2	212	8	1	147	1
pAPEC1990_61	8	7	265	1	263	263	265	265	265	265	265	263	7	7	8	263	262	8	1365	0	263	7	3	0	1	0	8	7	1	211	7	0	146	0
pAR060302	9	8	266	2	264	264	266	266	266	266	266	264	8	8	9	264	263	9	1366	1	264	8	4	1	0	1	9	8	2	212	8	1	147	1
pAR_0156_unitig_1	9	8	266	2	264	264	266	266	266	266	266	264	8	8	9	264	263	9	1366	1	264	8	4	1	2	1	9	8	2	212	8	1	147	1
pASP-a58	9	8	266	2	264	264	266	266	266	266	266	264	8	8	9	264	263	9	1366	1	264	8	4	1	2	1	9	8	2	212	8	1	147	1
pBML2526	215	214	420	216	418	418	420	420	420	420	420	418	214	214	215	418	419	215	1338	215	418	214	218	215	216	215	215	214	216	76	214	215	156	215
pCFSAN000405_01	8	7	265	1	263	263	265	265	265	265	265	263	7	7	8	263	262	8	1365	0	263	7	3	0	1	0	8	7	1	211	7	0	146	0
pCFSAN000934_02	9	8	266	2	264	264	266	266	266	266	266	264	8	8	9	264	263	9	1366	1	264	8	4	1	0	1	9	8	2	212	8	1	147	1
pCFSAN001921	60	59	245	53	243	243	245	245	245	245	245	243	59	59	60	243	242	60	1365	52	243	59	55	52	53	52	60	59	53	211	59	52	176	52
pCFSAN007425_01	9	8	266	2	264	264	266	266	266	266	266	264	8	8	9	264	263	9	1366	1	264	8	4	1	0	1	9	8	2	212	8	1	147	1
pCFSAN007428_01	8	7	265	1	263	263	265	265	265	265	265	263	7	7	8	263	262	8	1365	0	263	7	3	0	1	0	8	7	1	211	7	0	146	0
pCVM22425	9	8	266	2	264	264	266	266	266	266	266	264	8	8	9	264	263	9	1366	1	264	8	4	1	0	1	9	8	2	212	8	1	147	1
pCf52	9	8	266	0	264	264	266	266	266	266	266	264	8	8	9	264	263	9	1366	1	264	8	4	1	2	1	9	8	2	212	8	1	147	1
pCf53	9	8	266	0	264	264	266	266	266	266	266	264	8	8	9	264	263	9	1366	1	264	8	4	1	2	1	9	8	2	212	8	1	147	1
pDGO100	271	270	2	264	0	0	2	2	2	2	2	0	270	270	271	0	3	271	1426	263	0	270	266	263	264	263	271	270	264	410	270	263	374	263
pEA1509_A	2	1	273	9	271	271	273	273	273	273	273	271	1	1	2	271	270	0	1367	8	271	1	11	8	9	8	2	1	9	215	1	8	152	8
pECAZ155_KPC	3	2	274	10	272	272	274	274	274	274	274	272	2	2	1	272	271	3	1368	9	272	2	12	9	10	9	3	2	10	216	2	9	153	9
pEc19	9	8	266	0	264	264	266	266	266	266	266	264	8	8	9	264	263	9	1366	1	264	8	4	1	2	1	9	8	2	212	8	1	147	1
pEc78	9	8	266	0	264	264	266	266	266	266	266	264	8	8	9	264	263	9	1366	1	264	8	4	1	2	1	9	8	2	212	8	1	147	1
pEc9	9	8	266	0	264	264	266	266	266	266	266	264	8	8	9	264	263	9	1366	1	264	8	4	1	2	1	9	8	2	212	8	1	147	1
pEcl-Gr4873	1	0	272	8	270	270	272	272	272	272	272	270	0	0	1	270	269	1	1366	7	270	0	10	7	8	7	1	0	8	214	0	7	151	7
pHM881QN	8	7	265	1	263	263	265	265	265	265	265	263	7	7	8	263	262	8	1365	0	263	7	3	0	1	0	8	7	1	211	7	0	146	0
pIMP-PH114	8	7	265	1	263	263	265	265	265	265	265	263	7	7	8	263	262	8	1365	0	263	7	3	0	1	0	8	7	1	211	7	0	146	0
pIP1202	1	0	272	8	270	270	272	272	272	272	272	270	0	0	1	270	269	1	1366	7	270	0	10	7	8	7	1	0	8	214	0	7	151	7
pIP40a	272	271	7	265	5	5	7	7	7	7	7	5	271	271	272	5	2	272	1425	264	5	271	267	264	265	264	272	271	265	413	271	264	375	264
IncAC-LS6	9	8	266	2	264	264	266	266	266	266	266	264	8	8	9	264	263	9	1366	1	264	8	4	1	2	1	9	8	2	212	8	1	147	1
pKAZ1	3	2	274	10	272	272	274	274	274	274	274	272	2	2	3	272	271	3	1368	9	272	2	12	9	10	9	3	2	10	216	2	9	153	9
pKAZ2	3	2	274	10	272	272	274	274	274	274	274	272	2	2	3	272	271	3	1368	9	272	2	12	9	10	9	3	2	10	216	2	9	153	9
pKAZ3	4	3	275	11	273	273	275	275	275	275	275	273	3	3	4	273	272	4	1369	10	273	3	13	10	11	10	4	3	11	217	3	10	154	10
pKAZ4	271	270	2	264	0	0	2	2	2	2	2	0	270	270	271	0	3	271	1426	263	0	270	266	263	264	263	271	270	264	410	270	263	374	263
pKAZ5	16	15	281	17	279	279	281	281	281	281	281	279	15	15	16	279	278	16	1374	16	279	15	19	16	17	16	16	15	17	223	15	16	160	16
pKEC-a3c	0	1	273	9	271	271	273	273	273	273	273	271	1	1	2	271	270	2	1365	8	271	1	11	8	9	8	2	1	9	215	1	8	152	8
pKEC-dc3		1	273	9	271	271	273	273	273	273	273	271	1	1	2	271	270	2	1365	8	271	1	11	8	9	8	2	1	9	215	1	8	152	8
pKOX-86d	1		272	8	270	270	272	272	272	272	272	270	0	0	1	270	269	1	1366	7	270	0	10	7	8	7	1	0	8	214	0	7	151	7
pKP1-NDM-1	273	272		266	2	2	0	0	0	0	0	2	272	272	273	2	5	273	1427	265	2	272	268	265	266	265	273	272	266	412	272	265	376	265
pKp55	9	8	266		264	264	266	266	266	266	266	264	8	8	9	264	263	9	1366	1	264	8	4	1	2	1	9	8	2	212	8	1	147	1
pNDM-116-17	271	270	2	264		0	2	2	2	2	2	0	270	270	271	0	3	271	1426	263	0	270	266	263	264	263	271	270	264	410	270	263	374	263
pNDM-1_Dok01	271	270	2	264	0		2	2	2	2	2	0	270	270	271	0	3	271	1426	263	0	270	266	263	264	263	271	270	264	410	270	263	374	263
pNDM-KN	273	272	0	266	2	2		0	0	0	0	2	272	272	273	2	5	273	1427	265	2	272	268	265	266	265	273	272	266	412	272	265	376	265
pNDM-US	273	272	0	266	2	2	0		0	0	0	2	272	272	273	2	5	273	1427	265	2	272	268	265	266	265	273	272	266	412	272	265	376	265
pNDM102337	273	272	0	266	2	2	0	0		0	0	2	272	272	273	2	5	273	1427	265	2	272	268	265	266	265	273	272	266	412	272	265	376	265
pNDM10469	273	272	0	266	2	2	0	0	0		0	2	272	272	273	2	5	273	1427	265	2	272	268	265	266	265	273	272	266	412	272	265	376	265
pNDM10505	273	272	0	266	2	2	0	0	0	0		2	272	272	273	2	5	273	1427	265	2	272	268	265	266	265	273	272	266	412	272	265	376	265
pNDM15-1078	271	270	2	264	0	0	2	2	2	2	2		270	270	271	0	3	271	1426	263	0	270	266	263	264	263	271	270	264	410	270	263	374	263
pP99-018	1	0	272	8	270	270	272	272	272	272	272	270		0	1	270	269	1	1366	7	270	0	10	7	8	7	1	0	8	214	0	7	151	7
pPG010208	1	0	272	8	270	270	272	272	272	272	272	270	0		1	270	269	1	1366	7	270	0	10	7	8	7	1	0	8	214	0	7	151	7
pPm14C18	2	1	273	9	271	271	273	273	273	273	273	271	1	1		271	270	2	1367	8	271	1	11	8	9	8	2	1	9	215	1	8	152	8
pR148	271	270	2	264	0	0	2	2	2	2	2	0	270	270	271		3	271	1426	263	0	270	266	263	264	263	271	270	264	410	270	263	374	263
R16a	270	269	5	263	3	3	5	5	5	5	5	3	269	269	270	3		270	1423	262	3	269	265	262	263	262	270	269	263	411	269	262	373	262
R55	2	1	273	9	271	271	273	273	273	273	273	271	1	1	2	271	270		1367	8	271	1	11	8	9	8	2	1	9	215	1	8	152	8
RA1	1365	1366	1427	1366	1426	1426	1427	1427	1427	1427	1427	1426	1366	1366	1367	1426	1423	1367		1365	1426	1366	1368	1365	1366	1365	1367	1366	1366	1336	1366	1365	1356	1365
pRM12581	8	7	265	1	263	263	265	265	265	265	265	263	7	7	8	263	262	8	1365		263	7	3	0	1	0	8	7	1	211	7	0	146	0
pRMH760	271	270	2	264	0	0	2	2	2	2	2	0	270	270	271	0	3	271	1426	263		270	266	263	264	263	271	270	264	410	270	263	374	263
pSCEC2	1	0	272	8	270	270	272	272	272	272	272	270	0	0	1	270	269	1	1366	7	270		10	7	8	7	1	0	8	214	0	7	151	7
pSD_174	11	10	268	4	266	266	268	268	268	268	268	266	10	10	11	266	265	11	1368	3	266	10		3	4	3	11	10	4	214	10	3	147	3
pSH111_166	8	7	265	1	263	263	265	265	265	265	265	263	7	7	8	263	262	8	1365	0	263	7	3		1	0	8	7	1	211	7	0	146	0
pSN254	9	8	266	2	264	264	266	266	266	266	266	264	8	8	9	264	263	9	1366	1	264	8	4	1		1	9	8	2	212	8	1	147	1
pSN254b	8	7	265	1	263	263	265	265	265	265	265	263	7	7	8	263	262	8	1365	0	263	7	3	0	1		8	7	1	211	7	0	146	0
pSRC119-AC	2	1	273	9	271	271	273	273	273	273	273	271	1	1	2	271	270	2	1367	8	271	1	11	8	9	8		1	9	215	1	8	152	8
pTC2	1	0	272	8	270	270	272	272	272	272	272	270	0	0	1	270	269	1	1366	7	270	0	10	7	8	7	1		8	214	0	7	151	7
pUMNK88	9	8	266	2	264	264	266	266	266	266	266	264	8	8	9	264	263	9	1366	1	264	8	4	1	2	1	9	8		212	8	1	147	1
pUO-STmRV1	215	214	412	212	410	410	412	412	412	412	412	410	214	214	215	410	411	215	1336	211	410	214	214	211	212	211	215	214	212		214	211	143	211
pVC1447	1	0	272	8	270	270	272	272	272	272	272	270	0	0	1	270	269	1	1366	7	270	0	10	7	8	7	1	0	8	214		7	151	7
pYDC637	8	7	265	1	263	263	265	265	265	265	265	263	7	7	8	263	262	8	1365	0	263	7	3	0	1	0	8	7	1	211	7		146	0
pYR1	152	151	376	147	374	374	376	376	376	376	376	374	151	151	152	374	373	152	1356	146	374	151	147	146	147	146	152	151	147	143	151	146		146
peH4H	8	7	265	1	263	263	265	265	265	265	265	263	7	7	8	263	262	8	1365	0	263	7	3	0	1	0	8	7	1	211	7	0	146	

Table S3. Pairwise SNP distance matrix calculated from a set of 67 IncC plasmids, including pUO-STmRV1, and the IncA plasmid RA1.
